# Supplementary material for: Bile Acids Induce Neurite Outgrowth in Nsc-34 Cells via TGR5 and a Distinct Transcriptional Profile
Source: Pharmaceuticals (Basel). 2023 Jan 24;16(2):174. doi: 10.3390/ph16020174 (PMC9963315; doi:10.3390/ph16020174)
Supplement: Supplementary file 1 [file pharmaceuticals-16-00174-s001.zip › pharmaceuticals-2120337-supplementary.pdf]

pharmaceutics

MDPI

Supplementary Materials

Bile Acids Induce Neurite Outgrowth in Nsc-34 Cells via TGR5 and a Distinct Transcriptional Profile

Hayley D. Ackerman [1,2] and Glenn S. Gerhard [1,*]

[1] Lewis Katz School of Medicine, Temple University, Philadelphia, PA 19140, USA
[2] Department of Molecular Oncology, H. Lee Moffitt Cancer Center and Research Institute, Tampa, FL 33612, USA
* Correspondence: gsgerhard@temple.edu; Tel.: +215-707-5415

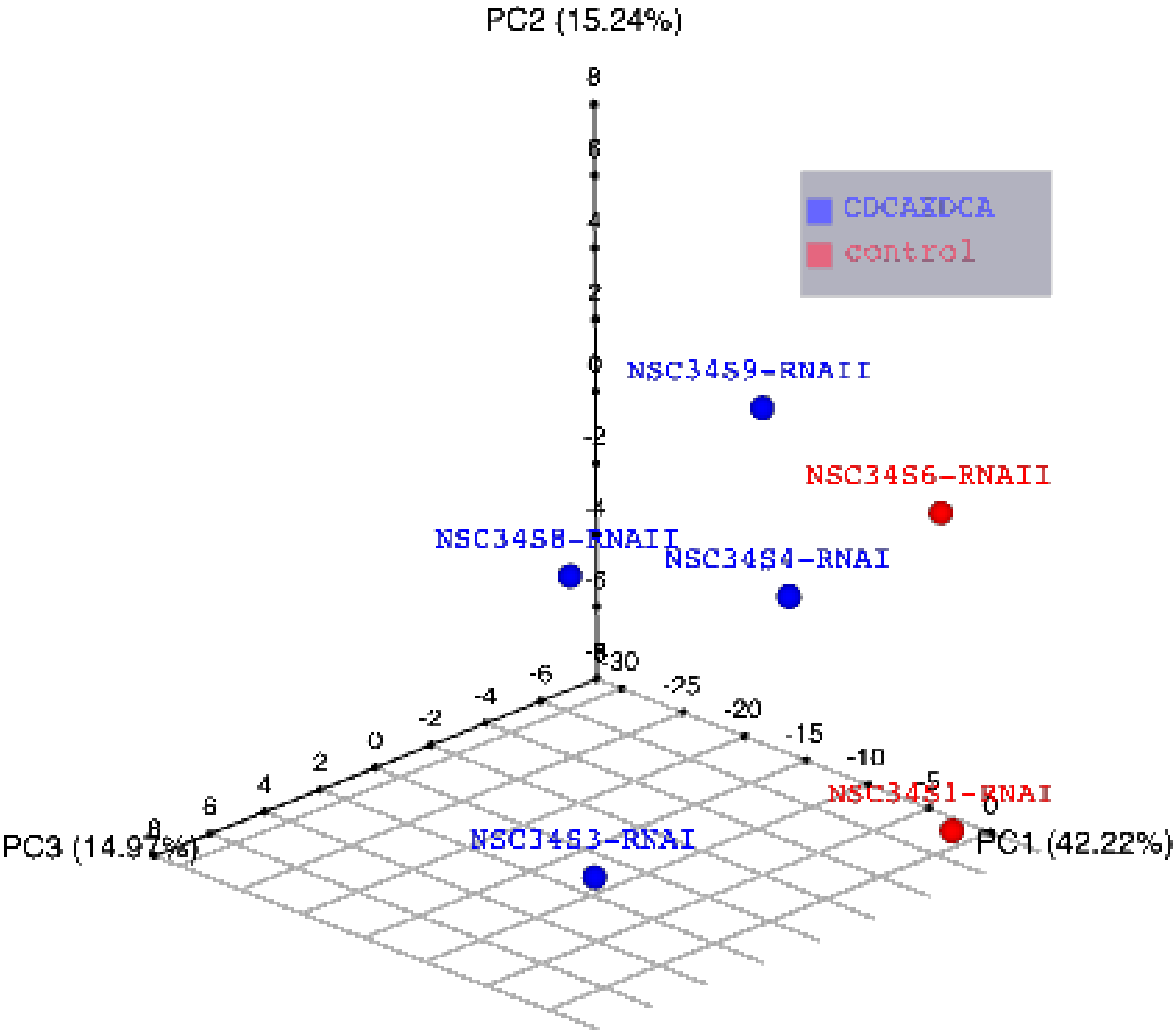

Figure S1. Principal component plot. The PCA plot shows the control basal medium (red) and DCA and CDCA (blue) samples in a three dimensional plot spanned by their first three principal components.

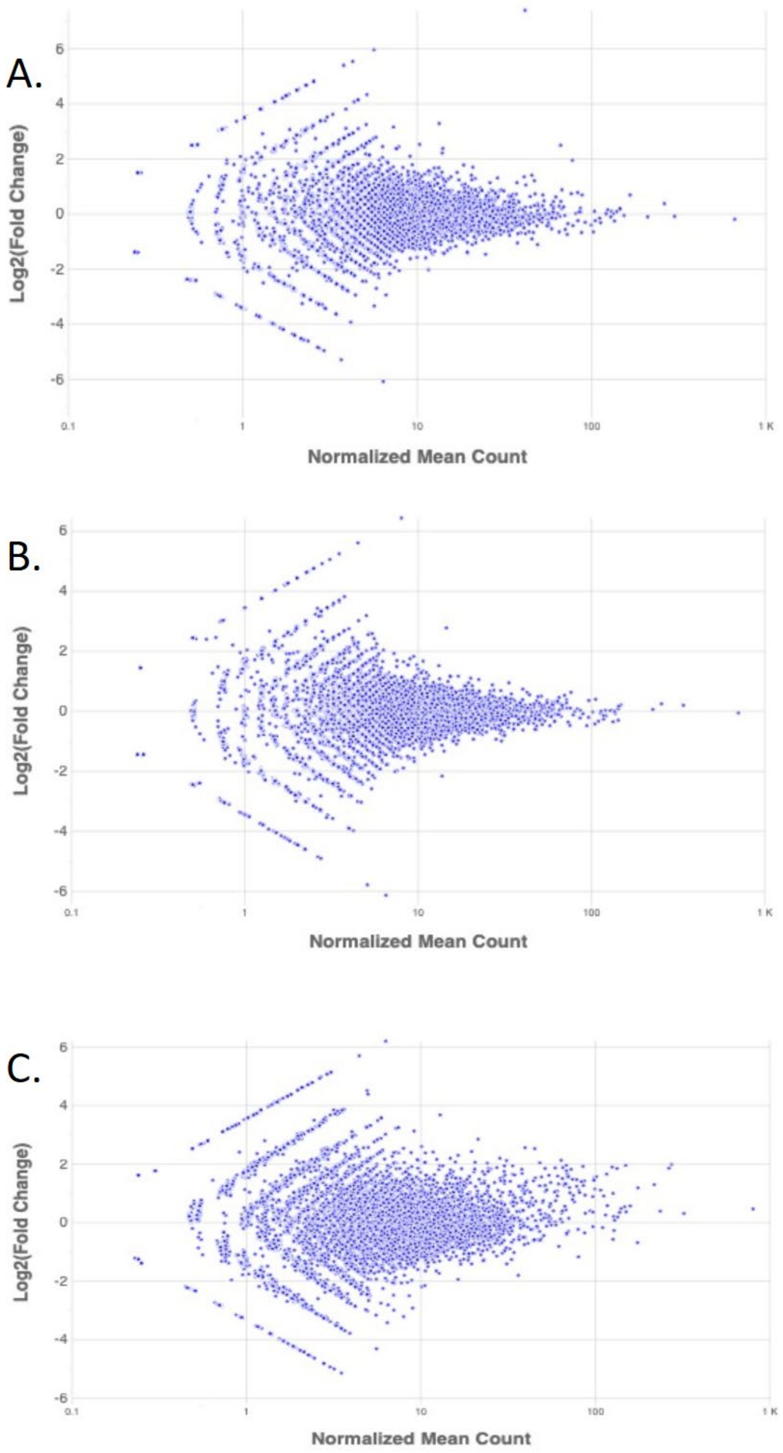

Figure S2. In DESeq2, the function $plotMA$ shows the log2 fold changes attributable to a given transcript over the mean of normalized counts for all the samples (**A**) Plot of RA versus DM. (**B**) Plot of DCA versus DM. (**C**) Plot of CDCA versus DM.

Table S1. Genes whose change in expression level relative to control basal neurite induction media lacking RA met a nominal ($p < 0.05$) level of statistical significance.

GeneName	log2FoldChange	*p*-Value	BASELINE	2 h
Dkk3	2.50	2.26E-13	19.92	112.97
Ctsb	1.96	4.41E-11	31.73	123.40
Cyp26b1	7.40	4.58E-07	0.49	82.52
Hid1	3.29	8.21E-05	2.48	24.24
Alpl	1.41	0.00018	24.80	65.97
Ubald2	−6.08	0.00040	12.59	0.19
Adnp	5.97	0.00061	0.18	11.12
Zbtb18	1.35	0.00061	23.16	58.98
Cd9	1.21	0.00079	28.36	65.52
Atp1a1	0.69	0.00084	126.90	205.38
Zfp445	2.22	0.0012	4.91	22.91
Rps2	−1.11	0.0014	66.99	30.98
Scg2	1.78	0.0019	8.34	28.62
Plxnd1	1.70	0.0020	9.84	31.85
Ikbip	5.54	0.0023	0.18	8.36
Lgals1	−1.46	0.0029	36.16	13.17
Irs2	1.31	0.0032	16.58	41.14
Gse1	1.14	0.0035	23.32	51.29
Ctsd	0.78	0.0036	61.12	105.22
Mcam	−5.29	0.0049	7.16	0.18
Ptp4a3	2.53	0.0050	3.05	17.59
Tnfsf13b	3.16	0.0057	1.47	13.16
Txnrd1	1.06	0.0058	27.19	56.71
Sdf4	2.38	0.0058	4.52	23.53
Hand2	0.88	0.0072	47.57	87.54
Wdr75	−2.02	0.0087	18.61	4.58
Chrna3	1.51	0.0089	8.80	25.06
Serinc1	0.69	0.0089	67.35	108.96
Stx7	1.41	0.0092	11.20	29.72
Tubb5	−0.67	0.0095	107.62	67.59
Stmn1	−1.05	0.0103	50.69	24.42
Cdh6	4.34	0.0109	0.48	9.82
Arhgap11a	−3.34	0.0118	10.32	1.02
Polr2m	−1.38	0.0119	27.94	10.73
Metrn	1.68	0.0120	6.34	20.24
Celf4	1.29	0.0126	12.47	30.47
Tshz2	1.14	0.0129	16.33	35.91
Cenpe	−2.94	0.0135	11.73	1.53
Mki67	−0.90	0.0137	59.13	31.76
Pum1	2.38	0.0137	2.46	12.76
Syt1	0.74	0.0138	57.22	95.75
Cerk	2.44	0.0141	2.45	13.26
Gramd1b	1.67	0.0148	5.99	19.09
Pcsk6	−1.42	0.0152	28.35	10.63
Ica1	−4.96	0.0155	5.67	0.18
Marcksl1	1.25	0.0161	12.16	28.97
Gabarapl1	4.15	0.0171	0.49	8.68
Fam102a	2.37	0.0174	2.45	12.65

H19	4.15	0.0174	0.49	8.67
Maml3	2.79	0.0181	1.47	10.15
Ptma	−0.65	0.0182	124.95	79.62
9330182L06Rik	2.10	0.0203	3.44	14.76
Vegfa	1.52	0.0203	7.16	20.53
Dcaf6	4.07	0.0208	0.49	8.22
Scd2	0.38	0.0211	227.39	295.52
Bmt2	2.74	0.0214	1.46	9.74
Fbxo38	−2.70	0.0226	10.10	1.55
Fyttd1	−1.43	0.0233	20.85	7.72
Tuba1b	−0.74	0.0240	90.52	54.28
Aurkb	−4.87	0.0240	5.31	0.18
Eif2ak3	3.25	0.0242	0.97	9.23
Hace1	2.45	0.0252	1.96	10.71
Glrx3	−4.84	0.0256	5.19	0.18
Ttc39b	1.57	0.0265	5.86	17.40
Ccndbp1	4.82	0.0266	0.17	4.93
Cog2	4.82	0.0266	0.17	4.93
Hpcal4	4.82	0.0266	0.17	4.93
Kcnn3	1.56	0.0269	5.88	17.33
Ephx1	1.24	0.0271	10.29	24.40
Kif2c	−3.93	0.0278	7.80	0.51
Prkaca	1.35	0.0278	8.81	22.45
Chka	2.66	0.0282	1.46	9.17
Tagln2	−1.59	0.0282	16.77	5.59
Plk1	−2.16	0.0284	11.37	2.54
Tmem41b	2.65	0.0286	1.47	9.23
Hsp90aa1	−0.65	0.0293	79.58	50.73
Ltbp3	1.44	0.0296	6.92	18.75
Rbfox2	1.52	0.0314	5.96	17.05
Ccna2	−1.41	0.0315	20.42	7.66
Ltbr	3.10	0.0318	0.96	8.23
Mvb12a	2.61	0.0331	1.50	9.13
Gstm2	1.28	0.0339	8.82	21.48
Cyth3	0.73	0.0345	35.72	59.16
Hoxd8	3.81	0.0359	0.52	7.27
Trove2	2.57	0.0360	1.47	8.73
Ski	1.55	0.0364	6.06	17.74
Vamp4	−1.57	0.0368	14.93	5.03
Racgap1	−1.04	0.0374	29.15	14.16
Rrm2	−2.94	0.0376	7.85	1.02
Nsg2	0.88	0.0382	21.47	39.58
Rnf166	2.94	0.0395	0.99	7.59
Syncrip	−1.32	0.0406	21.31	8.52
Gprasp2	1.45	0.0415	6.26	17.14
Tspan13	1.27	0.0422	8.29	19.97
Srp54a	4.67	0.0437	0.17	4.40
Epb41l2	−1.11	0.0450	28.26	13.12
Mdm1	−1.40	0.0450	17.61	6.67
Dlg4	1.45	0.0454	5.90	16.15
Prpf19	1.35	0.0454	7.39	18.90

Phf12	−2.30	0.0455	10.02	2.04
Phox2a	0.84	0.0460	22.52	40.23
Mcm6	−1.94	0.0469	11.77	3.06
Tiprl	−2.18	0.0475	9.77	2.16
Nckap1l	2.87	0.0483	0.98	7.16
Fbln1	−1.62	0.0485	13.75	4.48
Clk2	2.46	0.0487	1.48	8.17
Kars	−0.95	0.0488	31.35	16.20
Rnaseh2a	−4.61	0.0488	4.55	0.19
Sun1	−2.44	0.0490	8.55	1.58
Mmgt1	−2.18	0.0492	9.30	2.05
Ptprr	2.43	0.0492	1.56	8.41
Agl	2.95	0.0493	0.98	7.57

Table S2. Genes whose change in expression level relative to control basal neurite induction media lacking DCA met a nominal ($p < 0.05$) level of statistical significance.

GENE	log2 Fold Change	*p*-Value	BASELINE	2 h
Sdf4	2.78	5.25E-05	3.70	25.37
Dynlt1b	6.44	6.44E-05	0.18	15.83
Ubald2	−6.13	0.00038	12.85	0.18
Oaz1	−5.78	0.00098	10.00	0.18
Adnp	5.61	0.0015	0.18	8.81
Kdelr2	−2.16	0.0037	22.45	5.01
Gpsm1	5.06	0.0143	0.18	6.00
Ikbip	4.92	0.0173	0.18	5.40
Sulf2	−4.90	0.0182	5.30	0.18
Nlk	3.19	0.0184	0.99	9.05
Gprasp1	0.95	0.0200	22.72	44.00
Gm38431	−4.85	0.0238	5.10	0.18
Kif2c	−3.98	0.0239	7.96	0.50
Nova1	−2.01	0.0240	11.76	2.92
Bclaf1	−0.99	0.0291	40.27	20.24
Rab23	3.02	0.0297	0.99	8.06
Erlin1	−2.58	0.0298	9.23	1.54
Adi1	4.77	0.0298	0.18	4.85
Mvb12a	2.59	0.0299	1.54	9.31
Nanos1	−3.87	0.0312	7.39	0.50
Rbm6	−3.90	0.0319	7.48	0.50
Ttbk2	−3.03	0.0336	8.35	1.02
Gas2l3	−1.98	0.0344	12.07	3.06
Foxm1	−3.01	0.0345	7.96	0.99
Tprgl	3.82	0.0354	0.50	7.00
Pcdha8	4.76	0.0357	0.18	4.81
Rpl34	2.42	0.0358	1.75	9.39
Asf1b	−1.89	0.0358	12.94	3.50
Hdgfl2	1.75	0.0363	4.11	13.83
Ube2o	1.83	0.0384	4.14	14.71
Ctdnep1	2.55	0.0389	1.50	8.78
Ubr2	2.25	0.0389	2.00	9.52
Eif1	1.17	0.0400	10.47	23.56
Plekha1	1.10	0.0400	12.79	27.39

Klf13	4.63	0.0432	0.18	4.37
Atp8b2	1.93	0.0433	3.01	11.48
Man2a1	2.04	0.0434	2.47	10.13
Dcun1d2	4.62	0.0441	0.18	4.33
Ppib	3.70	0.0449	0.50	6.49
Tpm2	−4.60	0.0456	4.28	0.18
Rps12	0.63	0.0462	59.65	92.62
2310022A10Rik	4.65	0.0468	0.18	4.45
Lgals1	−0.93	0.0471	36.93	19.34
Bysl	2.60	0.0475	1.48	8.97

Table S3. Genes whose change in expression level relative to control basal neurite induction media lacking CDCA met a nominal ($p < 0.05$) level of statistical significance.

GENE	log2 Fold Change	*p*-Value	BASELINE	2 h
Fau	2.53	0.0017	13.28	76.47
Rps26	1.99	0.0019	109.55	436.55
Rpl18a	1.96	0.0033	60.76	237.17
Rps28	2.85	0.0039	5.18	37.30
Rpl36	2.56	0.0040	11.49	67.54
Rpl41	1.86	0.0042	113.87	413.33
Rpl32	1.92	0.0042	35.57	134.25
Oraov1	3.68	0.0046	1.86	23.90
Uba52	1.85	0.0051	57.68	207.98
Rpl35	2.13	0.0056	23.60	103.56
Rplp1	2.04	0.0061	18.75	77.21
Rpl21	1.96	0.0065	27.85	108.27
1700021F05Rik	5.70	0.0067	0.17	8.70
Rps24	1.90	0.0068	52.74	196.67
Rps15	1.88	0.0086	27.75	102.29
Rpl10a	1.82	0.0086	31.91	113.07
Rps17	1.77	0.0089	30.99	105.65
Rps3	1.57	0.0095	51.18	151.56
Rps12	1.66	0.0108	55.62	175.96
Rpl37a	2.43	0.0119	6.11	32.89
Rpl37	1.87	0.0123	25.23	92.31
Mki67	−1.80	0.0125	56.19	16.09
Rpl38	2.20	0.0153	10.13	46.54
Chchd10	2.12	0.0154	11.58	50.20
Prph	1.53	0.0156	50.41	145.98
Rps6	1.45	0.0172	52.83	144.07
Rps10	1.58	0.0191	34.91	104.67
Rpl14	1.54	0.0202	35.69	103.47
Lbr	−3.21	0.0206	14.37	1.55
Zc3h13	−5.14	0.0208	6.80	0.19
Rps29	2.03	0.0210	9.45	38.51
Rps8	1.82	0.0211	16.74	58.90
Rpl27a	1.51	0.0211	33.65	95.65
Crip1	3.13	0.0213	1.88	16.38
Rpl18	1.62	0.0214	26.62	81.57
Rpl17	1.85	0.0221	22.37	80.50
Med23	−2.92	0.0226	15.82	2.09

Rpl6	1.45	0.0236	52.23	142.93
Mrpl20	3.23	0.0250	1.40	13.13
Rps11	1.94	0.0250	12.42	47.73
Myef2	−4.31	0.0253	10.58	0.53
Minos1	2.06	0.0259	7.88	32.95
Rpl28	1.66	0.0263	33.00	104.61
Atp5h	1.88	0.0271	11.60	42.84
Rpl31	1.40	0.0274	45.71	120.33
Tprgl	4.39	0.0276	0.45	9.49
Aprt	2.30	0.0282	5.50	27.13
Josd2	5.14	0.0284	0.17	5.92
Ftl1	1.27	0.0287	50.10	120.54
Rpl4	1.30	0.0294	125.64	309.30
Gm1673	4.51	0.0295	0.41	9.40
Ikbip	5.13	0.0304	0.17	5.89
Rpl12	1.59	0.0305	30.32	91.26
Cebpb	5.10	0.0307	0.17	5.73
Gstp1	1.85	0.0309	10.59	38.24
Nenf	3.58	0.0309	0.91	10.86
Rpl19	1.48	0.0311	39.08	108.86
Emp1	−4.93	0.0325	5.83	0.19
Rpl27	1.53	0.0336	23.23	67.24
Rpl24	1.43	0.0336	46.65	125.75
Ovca2	5.07	0.0340	0.17	5.62
Bex3	1.94	0.0348	13.82	53.18
Gas2l3	−3.42	0.0349	11.72	1.09
Naxe	3.49	0.0350	0.92	10.37
Smc4	−1.94	0.0350	25.89	6.74
Pgls	2.20	0.0362	5.26	24.18
Top1	−2.99	0.0366	12.39	1.56
Uchl1	1.47	0.0371	41.49	114.66
Gm15772	1.83	0.0372	11.04	39.36
Rpl9	1.78	0.0372	11.13	38.21
Acbd6	2.38	0.0379	3.41	17.80
Rps4x	1.32	0.0386	38.35	96.02
Rpsa	1.19	0.0400	107.15	243.68
Pomp	2.41	0.0406	3.72	19.74
Ncan	−4.81	0.0412	5.38	0.19
Snx19	−4.81	0.0417	5.36	0.19
Rps20	1.56	0.0429	24.82	73.35
Pfdn5	2.07	0.0434	6.05	25.41
Marcksl1	1.65	0.0437	11.19	35.11
Ndufa12	1.65	0.0438	16.17	50.75
Rps19	1.63	0.0444	16.83	52.03
Rps25	1.33	0.0445	38.86	97.57
Bnip1	4.93	0.0447	0.17	5.08
Snrpd2	3.58	0.0450	0.92	10.94
Mpv17l2	2.60	0.0463	2.84	17.29
Rps3a1	1.27	0.0465	38.85	93.63
Ifi30	3.22	0.0469	1.39	12.91
Top2a	−1.41	0.0476	45.11	16.93

Rps18	1.75	0.0483	13.78	46.20
Dek	−2.50	0.0490	14.54	2.58
Cox6a1	1.68	0.0493	18.00	57.87
Rnf166	3.30	0.0494	0.92	9.10
Cfdp1	1.84	0.0495	7.48	26.72
Rpl23a	1.37	0.0496	30.93	79.89
